# Correlations between the Changing Levels of Tissue Plasminogen Activator and Adiposity Following Exercise-Induced Weight Loss

**DOI:** 10.3390/nu14235159

**Published:** 2022-12-04

**Authors:** Chao Zhang, Jonathan Salamon, Ren Zhang

**Affiliations:** 1 Biostatistics Shared Resource, Winship Cancer Institute of Emory University, 718 Gatewood Rd. NE, Atlanta, GA 30322, USA; 2 Center for Molecular Medicine and Genetics, Wayne State University School of Medicine, Detroit, MI 48201, USA

**Keywords:** fibrinogen, subcutaneous adipose tissues, tissue plasminogen activator, visceral adipose tissues, weight loss

## Abstract

Cardiovascular disease is a major threat to global public health. Tissue plasminogen activator (TPA) is a serine protease that dissolves blood clots, which can also lead to excessive bleeding. Fibrinogen (FIBR), a glycoprotein, is converted by thrombin to fibrin and then to a fibrin-based blood clot. Both TPA and FIBR levels in the blood are associated with an increased risk of coronary heart disease, and the levels of the two factors are also positively correlated with total adipose tissue amounts. Visceral and subcutaneous adipose tissues (VAT and SAT) can contribute differently to whole-body metabolism. In this study, we sought to assess: (1) the strength of the correlation between the changing levels of the two factors and the changing amounts of VAT/SAT during exercise-induced weight loss, (2) whether there is any difference between the two types of adipose tissues in terms of the correlation, and (3) which factor, TPA or FIBR, is more sensitive to changes in adiposity? For this study, we analyzed the data from the diabetes prevention program (DPP), in which the participants were divided into three groups, with one group undergoing a lifestyle change that involved maintaining a minimum of 7% weight loss with physical activity. We found that the basal amounts of VAT and SAT were correlated with TPA and FIBR levels. However, following weight loss, adiposity changes were strongly correlated with the changing levels of TPA, but not FIBR, for both men and women. Therefore, TPA, but not FIBR, is sensitive to changes in adiposity. Furthermore, regarding TPA, weight loss sensitized its correlation with SAT, but not VAT. This study shows how adipose tissues distinctively affect TPA and FIBR levels, two factors associated with cardiovascular disease and ischemic stroke.

## 1. Introduction

Cardiovascular disease is a major threat to global public health and the leading cause of global mortality for the last 15 years [1,2,3,4,5]. To develop treatments for and prevent cardiovascular disease, it is important to elucidate correlations between known and suspected risk factors under various treatment conditions [2]. 

Tissue plasminogen activator (TPA) is responsible for the activation of fibrinolysis within the vascular bed, where it converts plasminogen to the fibrinolytic enzyme plasmin [6,7,8]. An overabundance of TPA, and therefore plasmin, can lead to hyperfibrinolysis and subsequent excessive bleeding. Conversely, a reduction in TPA levels can result in fibrin accumulation, leading to the formation of atherosclerotic plaques [6,7,8]. Both TPA and its primary regulatory factor, plasminogen activator inhibitor 1 (PAI-1), have been strongly implicated as predictors of cardiovascular disease [9,10,11,12]. 

Fibrinogen (FIBR) is a 340 kDa glycoprotein, for which synthesis is drastically increased during stress and inflammation in the cardiovasculature [13]. Since FIBR is responsible for clot formation and platelet aggregation, improper regulation of FIBR production can lead directly to the formation of atherosclerotic plaques. Atherosclerotic plaque formation may be further exacerbated during inflammation [13,14,15,16]. Exercise and weight loss have been robustly linked to reduced inflammatory markers [17,18,19,20,21,22,23,24]. It has been shown that TPA and FIBR exhibit positive correlations with the amount of total body fat [6,25,26,27,28,29,30]. TPA levels were reduced following lifestyle changes in those who developed diabetes, and this reduction in TPA levels was largely explained by the weight loss that followed the lifestyle change [31]. However, to fully understand this phenomenon, some questions need to be further explored.

1. In people who lose weight through exercise, how strong are the correlations between changes in adiposity and changes in respective TPA and FIBR levels?
2. Is there a difference between changes in VAT and SAT in terms of the correlations to TPA or FIBR levels?
3. Is there a difference between TPA and FIBR in terms of these correlations, that is, which factor is more sensitive to the changing adiposity?

These questions present a challenge because analyzing changes in VAT and SAT requires CT scans prior to and following a weight loss program. However, the diabetes prevention program (DPP) has created a dataset that provides the opportunity to address these questions [6,32,33,34]. The DPP is a 27-facility, randomized clinical trial. The DPP compared the effects of pharmacological therapy to a physical lifestyle change, longitudinally. The lifestyle change involved maintaining a minimum of 7% weight loss, achieved by undergoing 150 min of moderate physical activity per week for the duration of 1 year [34]. In this study, we analyzed data compiled throughout the DPP study to examine the relationship between changes in the total amount of different kinds of fat tissues, and the changes in TPA and FIBR levels in the DPP participants.

## 2. Materials and Methods

### 2.1. Study Design

The DPP data, Archive version 2.1, were obtained from the National Institute of Diabetes and Digestive and Kidney (NIDKK) data repository. The DPP was a randomized, controlled clinical trial conducted in the United States from 1996 to 2001. Among the participants, 55% were Caucasian, and 45% were minority groups, including African American, Alaska Native, American Indian, Asian American, Latino, and Pacific Islander. The DPP examined changes on the rate of developing diabetes and related symptoms via different interventions. Interventions investigated by the DPP included intensive lifestyle changes as well as treatment with metformin versus a placebo. Determination of the amounts of SAT, VAT, and circulating TPA and FIBR levels were performed as previously described [31]. This study included 1037 participants. Of the participants, 685 were female and 352 were male. Subject numbers in the lifestyle, metformin, and placebo arms were 229, 223, and 233 female and 110, 126, and 116 male participants, respectively. The study protocol was approved by the Institutional Review Boards of Wayne State University, and all studies were carried out in accordance with the approved guidelines.

### 2.2. Statistical Analysis

Paired *t*-tests were conducted to compare differences of quantitative variables between the baseline and year-one values. One-way ANOVA was performed to compare differences in changes of quantitative variables among lifestyle, metformin, and placebo, and the LSD method was performed for multiple comparison tests. Spearman rank correlation was used to detect correlations between two variables. All statistical tests were performed using SAS 9.4 (SAS Institute, Cary, NC, USA), and *p*-values < 0.05 were considered statistically significant. The study protocol was approved by the Institutional Review Boards of Wayne State University School of Medicine and of the Detroit Medical Center, and all studies were carried out in accordance with the approved guidelines.

## 3. Results

### 3.1. Subcutaneous Adipose Tissue

We performed paired *t*-tests to compare the levels of each variable between the baseline and year-one checkup variables. As Table 1 shows, the amount of SAT was significantly reduced at one year for the lifestyle intervention group for both men (*p* < 0.001) and women (*p* < 0.001). SAT also decreased for the metformin group in both men (*p* < 0.001) and women (*p* < 0.001), but not in the placebo group for men (*p* = 0.884) or women (*p* = 0.150). We next examined changes in SAT levels comparing baseline and year-one levels among the lifestyle, metformin, and placebo arms by performing one-way ANOVA. For men, the average change in SAT level for lifestyle, metformin, and placebo arms was −60.4 ± 57.6, −14.0 ± 40.2, and −0.60 ± 39.7, respectively. The average change in SAT level was significantly different when we compared the lifestyle intervention to the pharmacological metformin intervention and the placebo group (*p* < 0.001). For women, the average SAT level change for the lifestyle, metformin, and placebo groups was −58.3 ± 75.4, −25.1 ± 54.1, and −4.9 ± 45.1, respectively. The average change in SAT level was significantly different when the lifestyle intervention group was compared to the pharmacological intervention metformin and placebo groups (*p* < 0.001).

### 3.2. Visceral Adipose Tissue

Compared to baseline levels, the year-one VAT level significantly decreased for the lifestyle intervention group (Table 1). The average decrease value was −44.1 ± 50.8 for men (*p* < 0.001) and −28.7 ± 43.5 for women (*p* < 0.001). For the metformin intervention group, the average change in VAT level between the baseline level and year-one significantly decreased (*p* < 0.006) in women and showed no significant change for men. When we compared the placebo group to the baseline level, the amount of VAT did not significantly change in men or women. We next examined the average change in VAT levels between the lifestyle intervention, the metformin intervention, and the placebo group. The average change in the VAT level for the lifestyle intervention group was a much greater decrease than either the metformin group or the placebo group for both men (*p* < 0.001) and women (*p* < 0.001). 

### 3.3. Tissue Plasminogen Activator

Compared to baseline levels, the level of TPA at year-one significantly decreased in the lifestyle intervention group for both men (*p* < 0.001) and women (*p* < 0.001) (Table 1). For the metformin intervention group, TPA levels also decreased in both men (*p* < 0.001) and women (*p* < 0.001). For the placebo group, TPA levels significantly decreased in women (*p* < 0.020), while there was no significant change between baseline and year-one for men. Next, we examined the average change in TPA levels among the lifestyle intervention, metformin intervention, and placebo groups. Average TPA levels were significantly lower for the lifestyle intervention group than the corresponding placebo group in both men (*p* < 0.001) and women (*p* < 0.001). 

### 3.4. Fibrinogen

**Figure 1:**
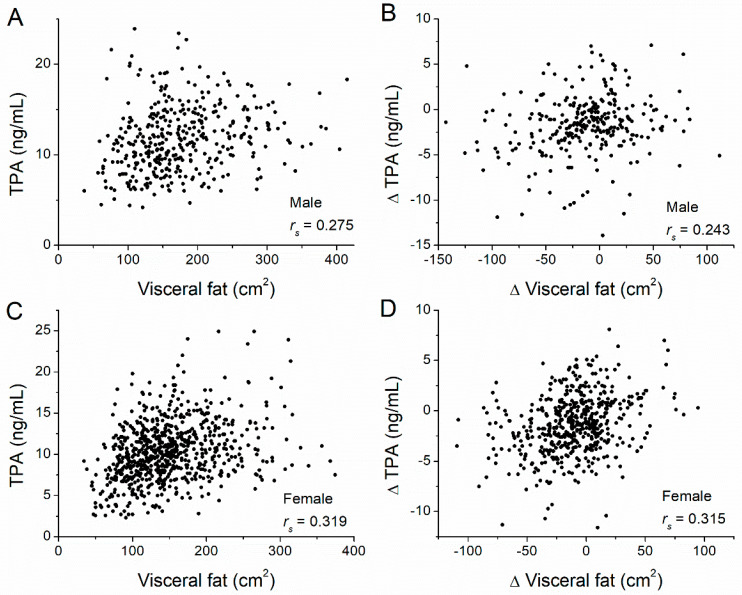
Correlations between visceral adipose tissue (VAT) volume and TPA levels, and between their changes. Correlations between (**A**) VAT and the TPA level (*r_s_* = 0.275, *p* < 0.001), and between (**B**) changes in VAT and changes in TPA levels (*r_s_* = 0.243, *p* < 0.001) in male participants of the diabetes prevention program. Correlations between (**C**) VAT and the TPA level (*r_s_* = 0.319, *p* < 0.001), and between (**D**) changes in VAT and changes in TPA levels (*r_s_* = 0.315, *p* < 0.001) in female participants. Adipose tissue volume was measured at baseline and the one-year follow-up by computed tomography at the L4–L5 level. Δ = value_(year 1)_ − value_(baseline)_, where values correspond to fat volume or TPA. The Spearman’s rank correlation coefficient, *r_s_,* was calculated.

**Figure 2:**
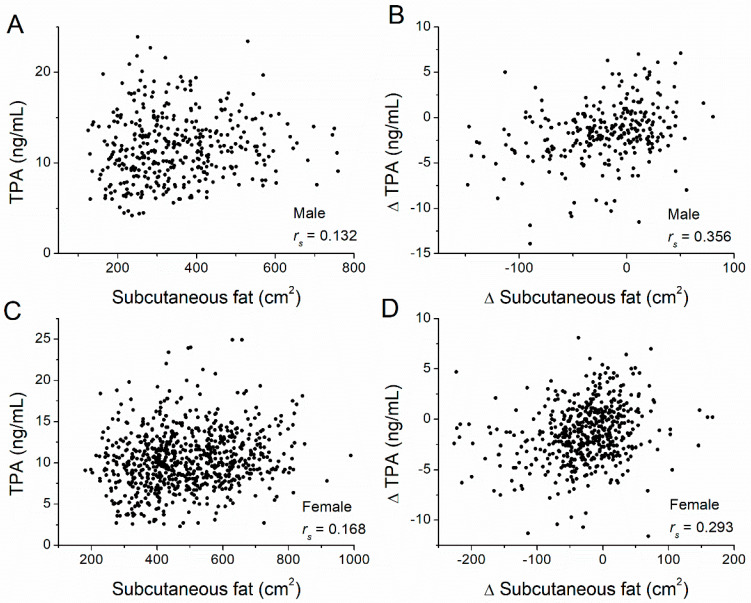
Correlations between subcutaneous adipose tissue (SAT) volume and TPA levels, and between their changes. Correlations between (**A**) SAT and the TPA level (*r_s_* = 0.132, *p* = 0.009), and between (**B**) changes in SAT and changes in TPA levels (*r_s_* = 0.356, *p* < 0.001) in male participants of the diabetes prevention program. Correlations between (**C**) SAT and the TPA level (*r_s_* = 0.168, *p* < 0.001), and between (**D**) changes in SAT and changes in TPA levels (*r_s_* = 0.293, *p* < 0.001) in female participants. Adipose tissue volume was measured at baseline and the one-year follow-up by computed tomography at the L4–L5 level. Δ = value_(year 1)_ − value_(baseline)_, where values correspond to fat volume or TPA. The Spearman’s rank correlation coefficient, *r_s_*, was calculated.

Table 1 shows that there was no significant difference in the FIBR level between the baseline and year-one levels for the lifestyle intervention, metformin intervention, and placebo groups in both men and women. We then examined the correlation between fat amounts at baseline and TPA levels at baseline for all participants (Table 2). In men, TPA was significantly and positively correlated with both VAT (*r_s_* = 0.275, *p* < 0.001) (Figure 1A) and SAT (*r_s_* = 0.132, *p* = 0.009) (Figure 2A). Similarly, in women, TPA was also significantly and positively correlated with both VAT (*r_s_* = 0.319, *p* < 0.001) (Figure 1C) and SAT (*r_s_* = 0.168, *p* < 0.001) (Figure 2C). Thus, for both men and women, TPA levels were significantly and positively correlated with both SAT and VAT. In addition, we examined correlations between the change in TPA levels and the change in VAT and SAT amounts (Table 3). In men, the change in TPA levels was significantly correlated with the change in both VAT (*r_s_* = 0.243, *p* < 0.001) (Figure 1B) and SAT amounts (*r_s_* = 0.356, *p* < 0.001) (Figure 2B). In women, the change in TPA levels was significantly correlated with the change in both VAT (*r_s_* = 0.315, *p* < 0.001) (Figure 1D) and SAT (*r_s_* = 0.293, *p* < 0.001) (Figure 2D). Therefore, for both men and women, changes in TPA levels were significantly and positively correlated with changes in both VAT and SAT amounts.

**Figure 3:**
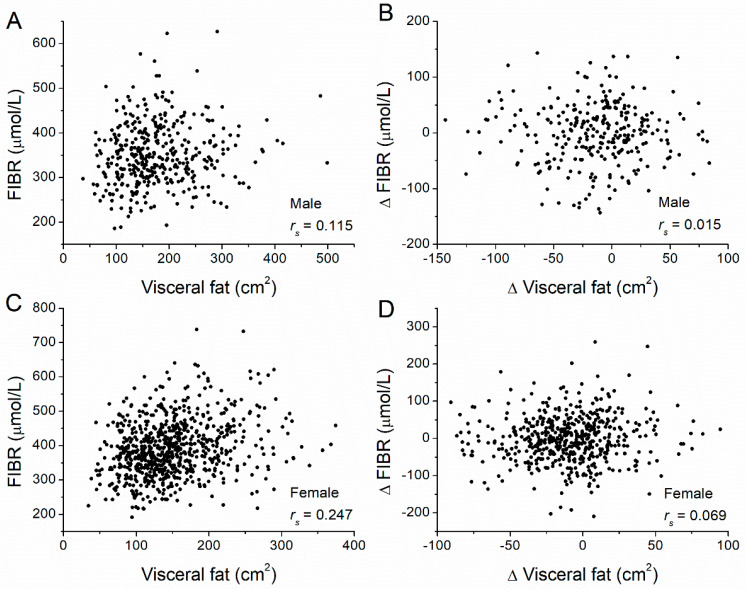
Correlations between visceral adipose tissue (VAT) volume and FIBR levels, and between their changes. Correlations between (**A**) VAT and the FIBR level (*r_s_* = 0.115, *p* = 0.024), and between (**B**) changes in VAT and changes in FIBR levels (*r_s_* = 0.015, *p* = 0.804) in male participants of the diabetes prevention program. Correlations between (**C**) VAT and the FIBR level (*r_s_* = 0.242, *p* < 0.001), and between (**D**) changes in VAT and changes in FIBR levels (*r_s_* = 0.069, *p* = 0.111) in female participants. Adipose tissue volume was measured at baseline and the one-year follow-up by computed tomography at the L4–L5 level. Δ = value_(year 1)_ − value_(baseline)_, where values correspond to fat volume or FIBR. The Spearman’s rank correlation coefficient, *r_s_*, was calculated.

**Figure 4:**
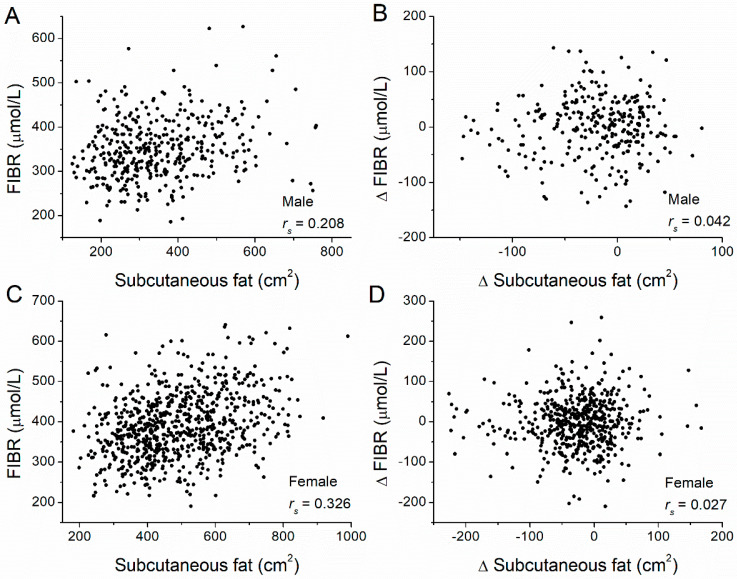
Correlations between subcutaneous adipose tissue (SAT) volume and FIBR levels, and between their changes. Correlations between (**A**) SAT and the FIBR level (*r_s_* = 0.208, *p* < 0.001), and between (**B**) changes in VAT and changes in FIBR levels (*r_s_* = 0.042, *p* = 0.475) in male participants of the diabetes prevention program. Correlations between (**C**) VAT and the FIBR level (*r_s_* = 0.326, *p* < 0.001), and between (**D**) changes in VAT and changes in FIBR levels (*r_s_* = 0.027, *p* = 0.543) in female participants. Adipose tissue volume was measured at baseline and the one-year follow-up by computed tomography at the L4–L5 level. Δ = value_(year 1)_ − value_(baseline)_, where values correspond to fat volume or FIBR. The Spearman’s rank correlation coefficient, *r_s_*, was calculated.

Next, we examined the correlation between the fat amount at baseline and FIBR at baseline for all participants (Table 2). In men, FIBR levels significantly and positively correlated with both VAT amounts (*r_s_* = 0.115, *p* = 0.024) (Figure 3A) and SAT amounts (*r_s_* = 0.208, *p* < 0.001) (Figure 4A). In women, FIBR levels also significantly and positively correlated with both VAT amounts (*r_s_* = 0.242, *p* < 0.001) (Figure 3C) and SAT amounts (*r_s_* = 0.326, *p* < 0.001) (Figure 4C). We conclude that FIBR levels significantly and positively correlate with both SAT and VAT amounts for both men and women. In addition, we also examined correlations between changes in FIBR levels and changes in VAT and SAT amounts. No significant correlation was observed between FIBR level changes with either changes to VAT amounts (Figure 3B,D) or SAT amounts (Figure 4B,D) for both men and women. 

### 3.5. Relationship between Changes in VAT and SAT Amounts and Changes in TPA Levels by Group

We then examined correlations between changes in the amount of adiposity and changes in the levels of TPA in the DPP participants. We examined the correlations in the lifestyle intervention, metformin intervention, and placebo groups (Table 4). For the lifestyle intervention group, the change in TPA levels was significantly and positively correlated with the change in both VAT amounts (*r_s_* = 0.477, *p* < 0.001) and the change in SAT amounts (*r_s_* = 0.319, *p* = 0.002) for men. This correlation was also observed in women for VAT amounts (*r_s_* = 0.390, *p* < 0.001) and SAT amounts (*r_s_* = 0.321, *p* < 0.001). For the metformin intervention group, no significant correlation was detected between the change in TPA levels for either VAT or SAT amounts in men. In women, the change in TPA levels was significantly correlated with the change in both VAT amounts (*r_s_* = 0.305, *p* < 0.001) and the change in SAT amounts (*r_s_* = 0.317, *p* < 0.001). For the placebo group, the correlation between changes in TPA levels and SAT amounts was significant (*r_s_* = 0.240, *p* < 0.001) only in men. No correlation between the change in TPA levels and VAT amounts was observed in either men or women.

### 3.6. Relationship between Changes in VAT and SAT Amounts and Changes in FIBR Levels by Group 

Next, we examined the correlation between changes in the size of different fat deposits and changes in FIBR levels for each group (Table 4). For the lifestyle intervention group, the change in FIBR levels was significantly and positively correlated with the change in both VAT amounts (*r_s_* = 0.207, *p* = 0.050) and SAT (*r_s_* = 0.228, *p* = 0.031) for men. In women, no significant correlation was detected between changes in FIBR levels and changes in either VAT or SAT amounts. For the metformin intervention group, changes in FIBR levels were negatively correlated with changes in VAT amounts in men (*r_s_* = −0.223, *p* = 0.022). However, changes in FIBR levels were positively correlated with changes in VAT amounts in women (*r_s_* = 0.162, *p* = 0.034). No significant correlation was observed in either men or women between changes in FIBR levels and changes in SAT amounts. For the placebo group, no significant correlation between changes in FIBR levels and changes in VAT or SAT amounts was observed for either gender.

## 4. Discussion

Heart disease and stroke are the first and fifth leading causes of death in the United States, respectively. Atherosclerosis is the buildup of fatty substances in the arteries, and it can lead to heart disease and stroke. TPA, as a medication that dissolves blood clots, is a life-saving treatment for both stroke and heart attack. Therefore, TPA is the most common treatment for ischemic stroke caused by a clot. FIBR, which is produced by the liver and activated during the clotting process, is a sign of thrombosis. It is important to clarify any correlation between adiposity and the levels of TPA and FIBR. 

Using the DPP data, we compared variations in TPA and FIBR levels to changes in visceral and subcutaneous adipose tissue amounts, following one year of lifestyle intervention or metformin treatment. We found that both the amounts of SAT and VAT, as well as the total change in SAT and VAT, were positively correlated with TPA level changes across the groups. Additionally, total SAT and VAT were positively correlated with FIBR levels. However, changes in SAT and VAT amounts did not appear correlated with changes in FIBR levels. 

**Figure 5:**
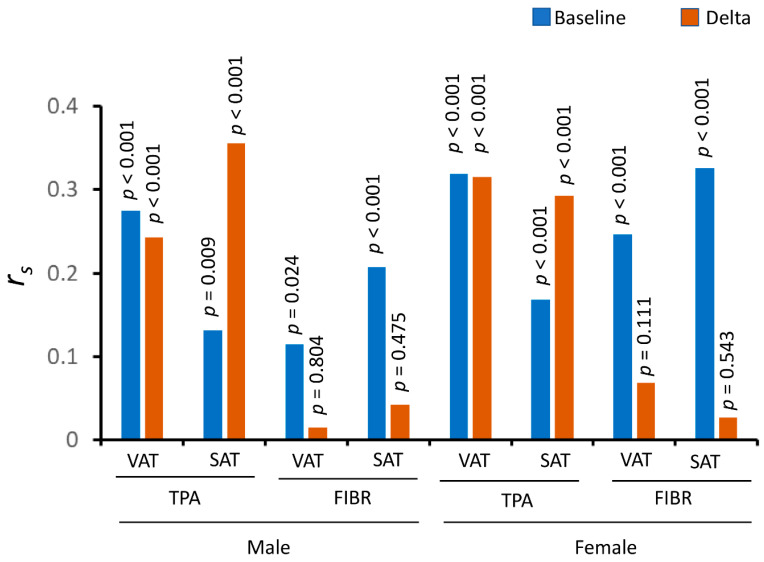
Correlation coefficients and *p*-values for the correlations between adiposity and levels of TPA and FIBR.

Comparing the lifestyle intervention group, which had a significant weight loss, with the placebo group, there were major differences between TPA and FIBR, or between VAT and SAT (Figure 5). For TPA, both baseline and changing levels of TPA were significantly correlated with adiposity. However, for FIBR, although baseline FIBR levels were significantly correlated with adiposity, its changing levels were not corrected with adiposity. This is true for both VAT and SAT, for both men and women. For example, the *p*-values for the correlations between changing FIBR and SAT were 0.475 and 0.543 in men and women, respectively, while the correlations for the baseline levels were highly significant (*p* < 0.001) (Figure 5).

We further examined the potential mechanisms that led to the dramatic differences between TPA and FIBR in their correlations with the changing adiposity. We hypothesized that it is likely to be due to the expression profiles of the two factors and to the roles of adipose tissues in affecting their circulating levels. Therefore, we examined the expression profiles among human tissues of the two factors. The Genotype-Tissue Expression (GTEx) database contains tissue-specific gene expression, determined using non-disease tissue samples from about 1000 individuals [35]. We examined the expression profiles of TPA and FIBR, which were determined by RNA-Seq, using the GTEx database (Supplementary Figure S1). TPA expression is ubiquitous; however, it is highly abundant in adipose tissues. In fact, the expression level of TPA in VAT is the second highest among the 54 tissues. In contrast, the FIBR expression is highly enriched in the liver. Therefore, it is likely that adipose tissues play an important role in determining the circulating levels of TPA, while for FIBR, the liver, rather than adipose tissues, is the determining factor for its circulating levels. 

Obesity is characterized by an accumulation of excessive adipose tissue, which is increasingly recognized as an endocrine organ that secretes a large number of circulating factors to regulate whole-body metabolism. These factors include leptin, adiponectin, plasminogen activator inhibitor-1, and inflammatory markers, such as tumor necrosis factor α and interleukin 6 [36]. Recent studies suggest that regional distribution of adipose tissue, e.g., VAT versus SAT, can differently affect the development of the metabolic syndromes [37]. It is possible that VAT and SAT have different secretion profiles in response to physiological and pathological signals. Therefore, although it was previously shown that weight loss has a durable effect on lowering TPA levels [6], here we focused on the difference between VAT and SAT in terms of the correlations. Although TPA levels were generally strongly associated with adiposity, there was also an important difference between VAT and SAT. Both VAT and its changes were strongly associated with TPA levels, however, weight loss sensitized the correlations between SAT and TPA levels. For instance, in men, the correlation coefficient was 0.132 (*p* = 0.009), between the baseline TPA and SAT, while the correlation coefficient became 0.356 (*p* < 0.001) between the changing TPA and SAT. This phenomenon also holds in women (Figure 5). Indeed, there was a clear depot difference in TPA expression between SAT and VAT (Supplementary Figure S1A). Therefore, the depot difference in the expression levels of TPA may be involved in how the circulating levels are affected by the weight loss.

Our study has some limitations. The correlation coefficients between basal adiposity and TPA levels and those between changing adiposity and TPA levels following weight loss were around 0.3, suggesting that the strength of the correlations was not strong, despite having statistically significant *p*-values. Additionally, correlation does not imply causation. That is, the changing levels of TPA were not necessarily the result of the changing adiposity. Other factors influenced by adiposity can affect TPA levels, or vice versa. Therefore, our study does not show a mechanism that explains these correlations. Furthermore, our study does not provide a mechanistical understanding of why adiposity is correlated with both TPA and FIBR, which appear to have opposing roles, because the former and the latter are involved in blood clot removal and forming, respectively.

## 5. Conclusions

We conclude that TPA levels are quite sensitive to changes in adiposity, and weight loss sensitizes the correlation between TPA levels and SAT, but not VAT. In contrast, although basal FIBR levels showed significant correlation with adiposity, the correlation between changes in adiposity and changes in FIBR levels was not statistically significant. This result shows that FIBR levels are not sensitive to changes in adiposity. This study shows how changes in adiposity distinctly affect the levels of TPA and FIBR, two factors associated with cardiovascular diseases and ischemic stroke.

## Figures and Tables

**Table 1:** Amounts of subcutaneous and visceral adipose tissues and levels of TPA and FIBR in the diabetes prevention program participants.

	N		Men			Women		
Group	(Male)	Variable	Baseline	Year 1	Delta	Baseline	Year 1	Delta
Lifestyle	339 (110)	Subcutaneous	347.1 ± 117.1	282.1 ± 108.6	−60.4 ± 57.6	488.0 ± 140.5	424.5 ± 138.8	−58.3 ± 75.4
		Visceral	181.2 ± 78.1	137.4 ± 67.5	−44.1 ± 50.8	150.4 ± 54.4	123.3 ± 53.8	−28.7 ± 43.5
		TPA (ng/mL)	12.0 ± 4.6	9.2 ±3.6	−3.0 ± 3.9	10.3 ± 3.5	8.5 ± 3.2	−1.9 ± 2.9
		FIBR (µmol/L)	360.7 ± 63.3	359.0 ± 66.3	−1.6 ± 60.7	391.2 ± 81.6	385.3 ± 82.2	−5.5 ± 61.8
Metformin	349 (126)	Subcutaneous	339.1 ± 128.2	334.4 ± 146.6	−14.0 ± 40.2	486.1 ± 145.6	445.4 ± 131.3	−25.1 ± 54.1
		Visceral	174.6 ± 70.0	172.6 ± 69.6	−5.6 ± 40.9	148.0 ± 58.6	138.9 ± 59.5	−5.6 ± 26.3
		TPA (ng/mL)	11.8 ± 4.6	9.9 ± 4.1	−2.0 ± 3.2	10.2 ± 3.5	8.4 ± 3.5	−1.7 ± 2.5
		FIBR (µmol/L)	353.9 ± 75.5	352.1 ± 77.2	0.9 ± 50.1	393.8 ± 77.4	396.4 ± 82.8	2.9 ± 69.3
Placebo	349 (116)	Subcutaneous	362.3 ± 136.4	359.6 ± 128.1	0.6 ± 39.7	487.4 ± 144.4	478.3 ± 142.3	−4.9 ± 45.1
		Visceral	173.9 ± 67.6	172.1 ± 66.3	−2.1 ± 33.5	150.4 ± 61.6	144.7 ± 52.6	0.4 ± 24.7
		TPA (ng/mL)	11.9 ± 3.7	11.7 ± 3.5	−0.6 ± 3.0	10.7 ± 3.6	10.2 ± 3.4	−0.5 ± 2.9
		FIBR (µmol/L)	353.8 ± 82.4	354.9 ± 73.4	−1.1 ± 63.2	400.0 ± 88.4	398.6 ± 87.3	−1.1 ± 67.6

**Table 2:** Correlation between baseline fat volumes and levels of baseline TPA and FIBR for all DPP participants.

		Men		Women	
	Fat Depot	*r_s_*	*p*-Value	*r_s_*	*p*-Value
TPA	Subcutaneous	0.132	0.009	0.168	<0.001
	Visceral	0.275	<0.001	0.319	<0.001
FIBR	Subcutaneous	0.208	<0.001	0.326	<0.001
	Visceral	0.115	0.024	0.242	<0.001

**Table 3:** Correlation between changes in size of different fat depots and changes in levels of TPA and FIBR.

		Men		Women	
ΔFat Depot	Markers	*r_s_*	*p*-Value	*r_s_*	*p*-Value
Subcutaneous	ΔTPA	0.356	<0.001	0.293	<0.001
	ΔFIBR	0.042	0.475	0.027	0.543
Visceral	ΔTPA	0.243	<0.001	0.315	<0.001
	ΔFIBR	0.015	0.804	0.069	0.111

**Table 4:** Correlation between changes in size of different fat depots and changes in levels of TPA and FIBR in the DPP participants by group.

			Men		Women	
	ΔFat Depot	Marker	*r_s_*	*p*-Value	*r_s_*	*p*-Value
Lifestyle	Subcutaneous	ΔTPA	0.319	0.002	0.321	<0.001
		ΔFIBR	0.228	0.031	0.124	0.103
	Visceral	ΔTPA	0.477	<0.001	0.390	<0.001
		ΔFIBR	0.207	0.050	0.030	0.692
Metformin	Subcutaneous	ΔTPA	0.178	0.070	0.317	<0.001
		ΔFIBR	−0.108	0.273	0.032	0.679
	Visceral	ΔTPA	−0.018	0.855	0.305	<0.001
		ΔFIBR	−0.223	0.022	0.162	0.034
Placebo	Subcutaneous	ΔTPA	0.240	<0.001	0.097	0.204
		ΔFIBR	0.149	0.148	−0.076	0.315
	Visceral	ΔTPA	0.155	0.134	0.078	0.306
		ΔFIBR	0.084	0.420	0.023	0.761

## Data Availability

The request for the original DPP data should be submitted to the NIDKK data repository. Secondary analysis results in the current study can be requested from the corresponding author.

## Supplementary Materials

The following supporting information can be downloaded at: https://www.mdpi.com/article/10.3390/nu14235159/s1, Figure S1. The expression profiles of (A) TPA and (B) FIBR among human tissues. The expression data was obtained from the GTEx database.

## Abbreviations

DPP: diabetes prevention program; FIBR, fibrinogen; SAT, subcutaneous adipose tissue; TPA, tissue plasminogen activator; VAT, visceral adipose tissue.
