# Supplementary material for: Correlations between the Changing Levels of Tissue Plasminogen Activator and Adiposity Following Exercise-Induced Weight Loss
Source: Nutrients. 2022 Dec 4;14(23):5159. doi: 10.3390/nu14235159 (PMC9741073; doi:10.3390/nu14235159)
Supplement: Supplementary file 1 [file nutrients-2041340-supplementary.pdf › nutrients-2041340-supplementary.pdf]

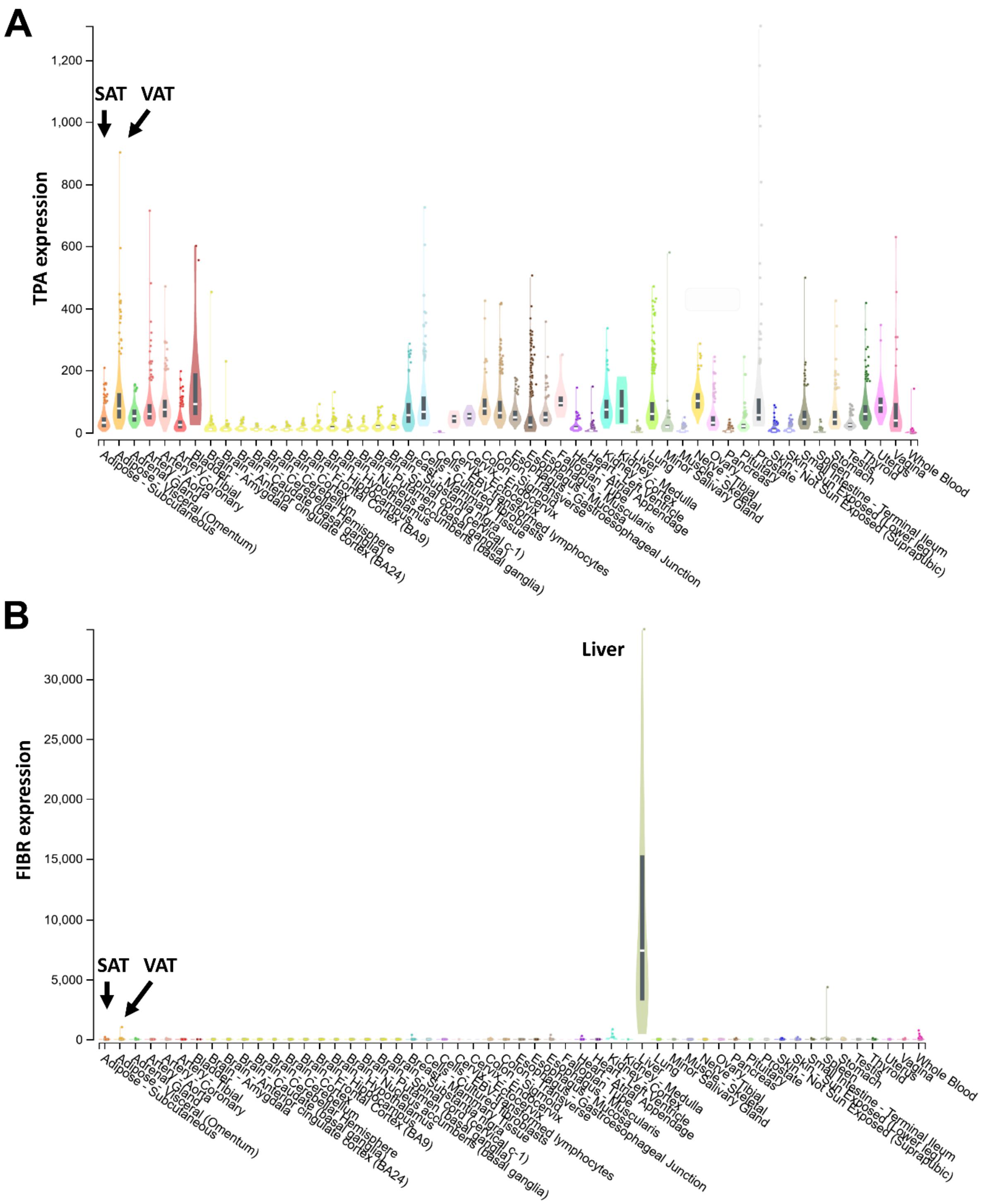

Supplemental Figure S1. The expression profiles of A) TPA and B) FIBR among human tissues. The expression data was obtained from the GTEx database.
